# The Impact of Forever Chemicals on Protein Structure and Function

**DOI:** 10.3390/ijms27052265

**Published:** 2026-02-28

**Authors:** Ethan J. Hasenoehrl, Will Kelly, Kenneth Kwansa-Aidoo, James Larson, Monika Tokmina-Lukaszewska, Robin Das Sourab, Robert A. Walker, Brian Bothner

**Affiliations:** Department of Chemistry and Biochemistry, Montana State University, Bozeman, MT 59717, USA; ethanhasenoehrl@montana.edu (E.J.H.); will.kelly@student.montana.edu (W.K.); kennethkwansaaidoo@montana.edu (K.K.-A.); james.larson16@montana.edu (J.L.); tokminalukas@montana.edu (M.T.-L.); robindas.sourab@student.montana.edu (R.D.S.)

**Keywords:** PFAS, proteins, albumins, hemoproteins, receptors, per- and polyfluoroalkyl substances, forever chemicals

## Abstract

Per- and polyfluoroalkyl substances (PFAS), commonly known as “forever chemicals,” are a category of manufactured compounds that have been widely used in applications such as firefighting foams, clothing, cookware, cosmetics, and food packaging since the 1940s. These chemicals are known to bioaccumulate in many species, including humans, with half-lives numbering years and decades. Many of these chemicals are already known for their acute and chronic adverse effects on human health, and the list of confirmed harmful outcomes has continued to grow quickly. Since PFAS are persistent in the environment and everyday products, the cumulative exposure risk is quite high. Recently, PFAS have come under regulatory scrutiny, with safe exposure limit guidelines being consistently lowered as detection methods continue to improve. The majority of the research cataloging the effects of PFAS on human health have, thus far, been concentrated around the development of reliable detection methods and mitigation strategies. Only recently have efforts shifted towards investigations of how PFAS affect biomolecular function in membranes and proteins. To aid future research on PFAS interactions with biomolecules, this review summarizes the current state of knowledge about PFAS impact on the structure and function of albumins, hemoproteins, nuclear receptors, and membrane receptors.

## 1. Introduction

Per- and polyfluoroalkyl substances (PFAS) are a class of molecules that contain a fluorinated alkyl chain of varying length. Water-soluble PFAS additionally contain a polar headgroup giving them amphiphilic properties. Because of these chemical properties, PFAS quickly have become essential to manufacturing, leading to widespread integration into countless products and applications. As of January 2026, there are nearly 15,000 PFAS compounds [1] that are widely used in applications such as food packaging, firefighting foams, water-repelling sprays and many more. However, PFAS’ exceedingly useful grease, water, and stain resistance properties have come at a cost. These molecules have been deemed “forever chemicals” because of their exceptional stability. This is due to the presence of strong carbon–fluorine bonds that resist chemical and biological breakdown in the environment. Therefore, since their proliferation beginning in the 1940s, PFAS have made their way into soil and drinking water [2], and eventually into the human body [3]. Estimates reported that by the year 2000, approximately 98% of the U.S. population had already been exposed [4].

**Figure 1:**
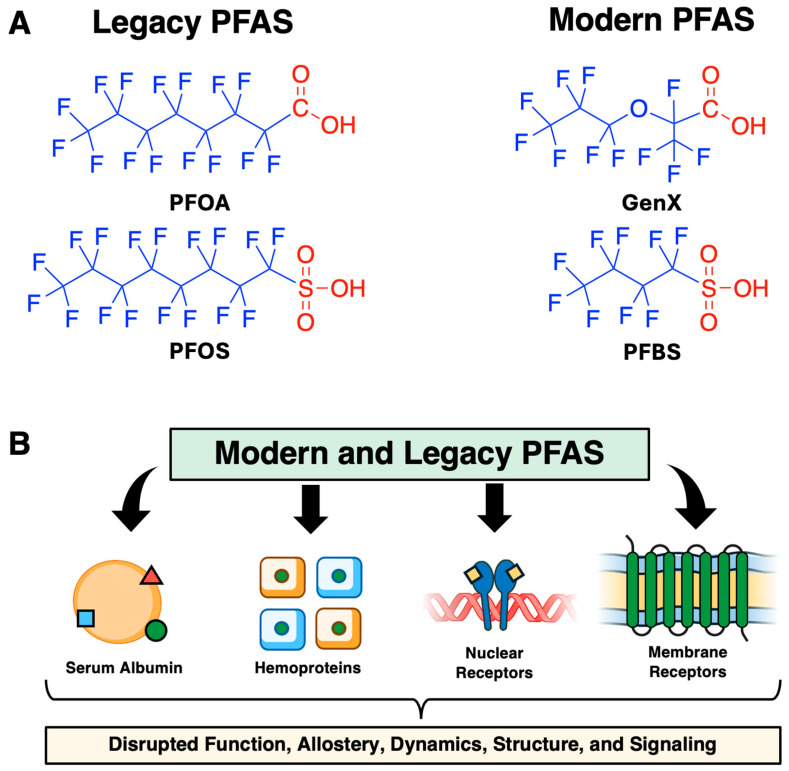
Structure and downstream effects of PFAS on proteins. (**A**) Molecular structure diagrams of perfluorooctanoic acid (PFOA), perfluorooctanesulfonic acid (PFOS), hexafluoropropylene oxide dimer acid (GenX), and perfluorobutanesulfonic acid (PFBS) are depicted. Non-polar tail regions and polar headgroup regions are depicted in blue and red, respectively. (**B**) Pictograms representing serum albumin, hemoproteins, nuclear receptors, and membrane are shown from left to right.

The main pathway for PFAS elimination from the human body is through urinary excretion. However, some of the compounds, such as perfluorooctanoic acid (PFOA) and perfluorooctanesulfonic acid (PFOS) (Figure 1), can bioaccumulate, with half-lives in serum being 2.7 and 3.4 years, respectively [5]. Studies have indicated a large variability in half-lives due to factors including sex, age, kidney function, and differences in exposure. As early as 1959, the acute and chronic effects on health associated with direct exposure to PFAS were well documented [6]. In 1979, a report documented that three out of nine pregnant factory workers gave premature birth, and some of the children were born with defects after exposure to PFAS. Another factory’s report from the same year revealed that dogs encountering PFAS compounds (ingestion of a single high dose) died within 48 h. Every year, the list of known adverse effect of PFAS on human health continues to grow. The most common outcomes include thyroid and kidney cancer, liver toxicity, decreased lung function, male infertility, hormonal imbalance in newborns and premature birth. In recent studies, PFAS exposure has been shown to be correlated to numerous conditions, such as elevated biomarkers of cardiovascular disease [7] and potential neurotoxic effects [8].

Current PFAS regulation by the U.S. Environmental Protection Agency (EPA) has set limits for PFOA and PFOS in drinking water of 4 ppt [9]. These values have been consistently lowered as detection methods improve over time. In addition, compounds like PFOA [10] and PFOS [11] have been phased out and are now categorized as “legacy PFAS.” These molecules have since been replaced with alternative PFAS that remain in production, such as perfluorobutanesulfonic acid (PFBS) and hexafluoropropylene oxide dimer acid (GenX). Unfortunately, PFBS and GenX have already been identified as having potential adverse effects. For example, studies have shown that GenX causes enlargement of mice livers [12], and abnormalities in female mouse development [13]. Even though some PFAS compounds are no longer manufactured in the U.S. and many other countries, their presence will continue to pose risks due to their long-lasting persistence in the environment, hazardous properties, and widespread historical use. The impact of these chemicals is therefore relevant and continues to reveal that there are many unexpected effects impacting public and environmental health.

To complement this rapidly developing field of toxicological research, structural biology-focused research has begun identifying the impacts of PFAS on proteins in terms of toxicodynamics. These compounds can bind to a wide range of proteins, like serum albumin [14] and hemoglobin [15]. PFAS have been shown to disrupt general protein structure and substrate binding in proteins such as milk protein β-lactoglobulin [16]. What remains unclear for most proteins disrupted by these molecules is the mechanism of action.

Since the seminal publication of the Monod–Wyman–Changeux model of allostery in the 1960s [17], allostery has emerged as an increasingly important aspect of therapeutic intervention and the mechanistic understanding of proteins in general. Allostery, literally meaning “other site,” is the phenomenon of effectors binding to a non-active site in a protein, causing a conformational change, and potentially affecting protein function. How PFAS impact allostery as well as other structure–function attributes of proteins is an understudied and important research topic as well as a critical concern for public health.

To aid in the development of this understanding, we will examine and review the current state of the literature regarding the impact of PFAS on the allostery, structure, and dynamics of proteins. Current research has been largely divided into four broad categories: albumin proteins, hemoproteins, nuclear receptors, and membrane receptors. In this review, we will first focus on hemoproteins and albumin proteins. These two categories of transport proteins are interesting to study because of their potential to bioconcentrate PFAS into biological material. Secondly, the impact of PFAS on the structure and signaling of membrane and nuclear receptor proteins will be investigated. Receptors in these classes are involved in far-reaching signaling cascades that have large impacts on homeostasis and general health. All four of these classifications of proteins will be examined in context with the PFAS-related research that has been undertaken and to serve as a reference for future structural biology studies.

## 2. Methods

A literature screening of PFAS–protein studies has distinguished three research phases. In the early 2000s, reports presented a proof of numerous PFAS binding to blood components, with a strong indication that albumins were the primary target. In the next stage, research progressed to characterize PFAS binding to proteins, with studies focusing on binding stoichiometry and PFAS–protein affinity. An important point worth noting is that over two decades of PFAS research has led to considerable discrepancies in the reported stoichiometry and binding affinity of these pollutants to proteins in general. Our review of these reports leads us to believe that the origin of these differences is related to tested PFAS concentration ranges, animal species and analytical methods used for investigation. More recently, the implementation of high-resolution/sensitivity analytical tools has allowed for more detailed characterization of the nature of PFAS interactions with proteins, including impact on the protein structure and mechanism of action on a molecular level.

A wide variety of quantitative techniques has been used to characterize PFAS–protein interactions. One such method is calorimetry, including the techniques of isothermal titration calorimetry (ITC) and differential scanning calorimetry (DSC) [18,19,20,21,22,23,24]. ITC and DSC both provide information about binding affinity and enthalpy, Gibbs free energies associated with PFAS binding, and changes to a protein’s heat capacity and structural stability. However, these techniques have limited ability to provide information on PFAS’ binding mechanism.

Optics-based techniques such as circular dichroism (CD) and UV-Vis spectroscopy can be utilized to study the interaction between PFAS and proteins. CD is a valuable technique for gaining information on changes to proteins’ secondary structure in solution. Additionally, changes in UV-Vis absorption can provide information on PFAS–protein interactions through mechanisms like static quenching of tryptophan residues [25,26,27]. Both CD and UV-Vis spectroscopy require high concentrations of protein and have lower sensitivity compared to other techniques. Other optical techniques like fluorescence [28,29,30,31], fluorescence quenching (FQ) [32,33], and differential scanning fluorimetry (DSF) [21,34,35] can be used to study how PFAS affect different proteins, probing hydrophobic interactions. However, the specificity of these techniques is limited, and obtaining information on the location of PFAS molecules within or adsorbed to proteins is not possible. Because of the hydrophobic nature of PFAS tails, DSF readings can be significantly impacted by interacting with the DSF dyes.

Structural biology approaches like X-ray crystallography or cryogenic electron microscopy can provide valuable information on PFAS binding sites and stoichiometry or large-scale conformational changes upon binding to different PFAS molecules [36,37,38]. While both methods excel in providing structural information on the single amino acid resolution, X-ray crystallography reports only on the most stable conformation due to the nature of crystallization. Cryogenic electron microscopy provides more information on in-solution behavior, yet deciphering specific dynamics is often challenging, generally requiring additional experiments. The biggest drawback of these otherwise excellent analytical tools is the extremely low throughput.

Mass spectrometry (MS) has been the predominant form of detecting PFAS in the environment from regulatory bodies due to its exceptional sensitivity (detection limit on parts-per-trillion or ppt levels). MS, especially electrospray ionization, can provide information on protein–ligand stoichiometry, binding site(s), affinity binding, complex stability and protein dynamics. MS coupled with ion mobility measurements can detect changes in protein structure as small as 5%; therefore, it is useful to probe the conformational ensemble, if present in solution [39]. In addition, MS requires low amounts of sample and has better throughput than other analytical tools described in this manuscript. The main drawback is correlating gas-phase results with in-solution behavior. As with all the other techniques, MS is strengthened when coupled with other approaches.

Computational approaches have become an accepted and useful tool for understanding protein structure, dynamics, ligand binding and allostery. Molecular docking is used to predict how a ligand will bind to a protein, often providing a binding affinity for a specific ligand–protein orientation [40]. Molecular dynamic simulations predict how proteins within a biomolecular system (protein, water, lipid bilayers, etc.) move by subjecting every atom in the system to force fields [41,42]. Force fields are fit to quantum mechanical calculation results and often to experimental measurements to improve the accuracy of the computational prediction. These *in silico* approaches range in necessary computing power, which is directly proportional to the size and complexity of the system simulated, time frame of the simulation, and complexity of force fields applied. The unquestionable advantage of computational methods is an excellent throughput allowing for screening hundreds of PFAS–protein interactions.

In this review, we provide an overview of the PFAS–protein interaction studies that investigated how “forever chemicals” affect the structure, function, and allosteric regulation of different protein classes.

## 3. Albumin

Serum albumins are the most abundant soluble proteins in the circulatory system. They are primarily responsible for maintaining osmotic pressure and pH of the blood as well as the transport of various exogenous and endogenous components from the blood to different tissues and organs [43]. In the past two decades, numerous reports have confirmed that some PFAS bind to albumins [23,25,28,29,36,44,45,46,47,48,49,50,51]. The formation of strong albumin–PFAS complexes significantly alters the biodistribution, elimination of half-life, and toxicity of these pollutants [24,52,53]. For example, PFOS can displace the variety of steroid hormones bound to serum proteins in birds and fish [54], decreasing vitamin B2 binding to human serum albumin (hSA) by 30% [24]; due to PFOA binding to bovine serum albumin (BSA), decreased oxygen reactive species formation was observed [55]. Warfarin, a drug used to prevent blood clot formation, decreases PFOS/PFOA binding to bovine and human serum albums [32].

In general, PFAS affinity for serum albumins varies greatly and depends on the length of polyfluorinated carbon chain, headgroup, type of isomer, and animal species being examined. Most PFAS compounds exist as linear and branched isomers [56]. The isomeric distribution of PFAS in the environment depends on the contamination source, mitigation methods, and varying physicochemical properties imparted by their chemical structures. The higher polarity of the branched isomers allows for their accumulation mostly in water, while linear isomers are more often found in soils. Bioaccumulation also shows species-specific preferences. For example, in contrast to humans, other animals tend to preferentially accumulate linear forms of PFOS/PFOA, as indicated by higher affinity binding to serum albumin [57,58,59,60,61,62]. These results should be considered in the context that the PFOS abundance in the environment may affect isomer distribution in humans, since PFOS degrades preferentially to branched forms [63,64].

Several published accounts have reported that albumins, hSA and BSA in particular, can bind single as well as multiple PFAS monomers, with binding affinities ranging from 10^−2^ to 10^−6^ M [28,32,45,47,48,62,65,66,67]—for example, PFOS:BSA 1:1 [54], PFOS:hSA 45:1 in PFOS-saturating conditions [24], predicted hSA maximum binding sites: 9 to PFOA and 11 to PFOS [28,49], and PFOS and PFOA still binding multiple monomers to albumins even in sub-stoichiometric amounts: PFOS/PFOA: BSA/hSA 1-8:1 [32]. The affinity binding depends strongly on the binding site location. For example, hSA Sudlow’s I drug binding site favors short-chain PFAS, such as perfluorobutanoic acid (PFBA) or Perfluorobutanesulfonic acid (PFBS) (C4), while the tryptophan site has higher affinity binding for long-chain PFAS such as perfluorododecanoic acid (PFDoA) (C12). On the other hand, hSA Sudlow’s II drug binding site shows a certain degree of exclusion for the sulfonate headgroup, regardless of the carbon chain length [28]. These observations were further confirmed by studies that used a broader spectrum of PFAS compounds testing the variable length of the carbon chains (4–13) and headgroup (carboxylic acids, sulfonic acids, and sulfonamides) [68,69,70]. The authors concluded that smaller PFAS compounds (up to 8 carbons) fit well into BSA/hSA binding pockets; therefore, the binding event is driven by two mechanisms: electrostatic interactions (polar headgroup) and hydrophobic effects (nonpolar C−F chain). For the larger PFAS molecules (over 8 carbons), the binding event is more complex due to limited accessibility to the albumins’ binding pockets caused by the reduced molecular flexibility of the C–F chain.

Hydrophobic interactions between serum albumins and PFAS have been examined using two different optical techniques: FQ and DSF [34,65]. Using the quenching behavior and resulting emission maximum blue shift of BSA’s two native tryptophan residues, the authors of these studies concluded that five different PFAS (including PFOA, Perfluorodecanoic acid (PFDA), PFOS and others) all showed evidence of specific binding within the protein’s hydrophobic cavity and the creation of a more hydrophobic environment around the tryptophan residues [65]. The study utilized three models: Stern–Volmer (K_SV_), modified Stern–Volmer (K_MSV_) and the Hill equation (K_Hill_, n_Hill_), the last of which was critical for revealing binding cooperativity (n_Hill_ > 1). Subsequent studies using DSF monitored changes in fluorescence emission as a function of temperature during thermal scans, again exploiting the tryptophan residue as an intrinsic fluorophore [34,71]. DSF uses the protein melting temperature (T_m_) to evaluate if ligand binding causes a stabilization effect (ΔT_m_ increases) that correlates with binding affinity [34]. For accurate results with serum albumins, the authors noted that analyzing DSF data was not trivial and required specific data transformation, truncation, smoothing, and normalization due to high background fluorescence. Dissociation constants (K_D_) derived from DSF were interpreted as relative binding affinities [71]. Combining the analysis of T_m_ stabilization and association constants from both equilibrium-based models and the Hill adsorption model confirmed that trends in PFAS binding affinity are collectively driven by factors including hydrophobicity (as inferred from the oil–water partitioning coefficient, log K_ow_) and steric effects [34,65].

**Figure 2:**
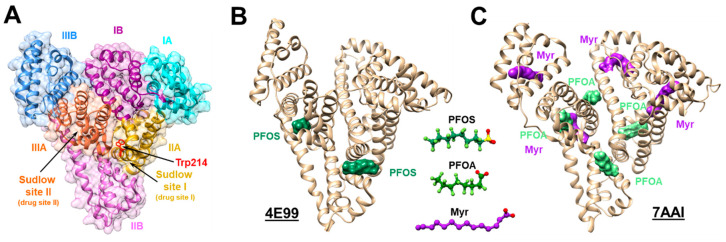
Crystal structures of PFOA and PFOS bound to human serum albumin (hSA). (**A**) Highlighted colors indicating subdomains with common binding sites; (**B**) hSA with two PFOS molecules bound in the absence of fatty acids, PDB ID: 4E99 [36]; (**C**) hSA with two PFOA molecules bound in the presence of myristic acids (Myr), PDB ID:7AAI [37].

Based on a structural model from X-ray crystallography (PDB ID: 4E99), the stoichiometry of PFOS binding to hSA was determined to be 2:1 [36] (Figure 2A,B). One PFOS monomer was found in the Sudlow site II drug binding domain, and the other one was in a fatty acid binding site located at the interface of subdomains IIA and IIB. The structure was obtained by incubating hSA in the presence of a 10-fold molar excess of PFOS. This was one of the first reports to provide information on PFAS impact on serum albumin structure. After binding two PFOS molecules, the complex became more compact (smaller solvent accessible surface area) in comparison to the unbound hSA. This result is quite different from the hSA–fatty acid complex behavior. The hSA’s “compactness” potentially increases protein complex stability and can therefore be partly responsible for the long plasma half-life of PFOS and other soluble PFAS species.

Subsequent studies reported that hSA and BSA can bind up to eight PFOA/PFOS molecules [32]. These results were obtained using native MS, fluorescence and molecular docking experiments. Unlike the conditions used to obtain the hSA crystal structure presented by Luo et al., 2012 [36] (PDB ID: 4E99) (Figure 2B), these solution-phase experiments were conducted at low micromolar PFAS concentrations that are much closer to PFAS concentrations found in the environment or in human blood/liver samples. Moreover, rather than using large PFAS excess, the protein:PFAS ratios were stoichiometric or lower. Interestingly, even for sub-stoichiometric PFAS amounts, data showed that both albumins could bind single PFOA/PFAS monomers. Another conclusion from these studies was that the tryptophan binding site might be the most favorable one for binding both PFOA and PFOS. Results also demonstrated conformational changes upon PFAS binding, with formation of the stable complexes having a high stoichiometric number of ligands. These results imply that strong interactions between PFOA/PFOS and serum albumins may affect albumin function and suggest a mechanism for PFAS toxicity.

A second crystal structure was obtained by incubating hSA in the presence of PFOA and a medium-length saturated fatty acid (PDB ID: 7AAI) [37] (Figure 2C). Structural characterization was supplemented with ITC to investigate the nature and locations of the PFOA binding sites. Based on the crystal structure, four PFOA binding sites were observed in the presence of myristic acid (Myr), a 14-carbon saturated fatty acid (C14:0). However, unlike in the case of PFOS–hSA interactions (hSA-Myr (PDB ID: 1EG7) vs. hSA–PFOS (PDB ID: 4E99)) [36], no significant structural difference was detected in comparisons between hSA–PFOA (PDB ID: 7AAI) and hSA–Myr (PDB ID: 7AAE) complexes. Despite their structural similarities, the binding mechanisms of PFOA and PFOS to hSA appear different (for example, a noticeable “elongation” of PFOS-bound hSA in comparison to PFOA–hSA in the presence of Myr). These studies also revealed that the PFOA binding sites on hSA have different affinities. There is one high-affinity binding site where interactions are facilitated by electrostatic and hydrophobic forces and three lower-affinity sites primarily maintained by hydrophobic interactions. Low-affinity-bound PFOA is sensitive to temperature change.

Serum albumins play an important role in the transportation and metabolism of a wide variety of endogenous and exogenous compounds, including drugs and nutrients, mostly through the formation of noncovalent complexes at specific binding sites in living organisms. Therefore, the structure and functional changes of serum albumin can affect the absorption, distribution, metabolism, and excretion of small molecules binding with the carrier protein. The elucidation of the molecular basis of the interaction between PFAS and hSA is expected to have implications for the development of superior hSA-based molecular receptors for diagnostic and biotechnological applications [72].

## 4. Hemoproteins

Hemoproteins are essential for a wide range of organisms, from microbes to mammals. They contain a characteristic heme prosthetic group that coordinates iron with a porphyrin ring. This cofactor endows proteins with the ability to perform essential biological functions. Some of the most ubiquitous hemoproteins are myoglobin (Mb), hemoglobin (Hb) and cytochromes. Mb and Hb are highly conserved diatomic gas carrier proteins, whereas cytochromes have diverse families that play key roles in many electron transfer systems.

Mb is a monomeric oxygen storage protein solely expressed in muscle tissues [73]. Mb contains one heme prosthetic group, allowing for a single oxygen molecule to bind and subsequently transfer to mitochondria in conditions of higher metabolic stress. In 2011, Qin et al. first reported that PFDA, a member of the PFAS family, significantly changed the heme environment to a more open conformation, exposing the binding site to water [74]. In the same study, PFOA and perfluoropentanoic acid (PFPA) did not induce a significant change in Mb fluorescence, indicating minimal structural changes near the heme group. The extra length of the hydrophobic tail of PFDA increases the molecule’s lipophilicity, and earlier work had shown that strongly lipophilic surfactants such as sodium dodecyl sulfate cause a similar “open” conformational change near the heme group [75].

More recent experiments complement this work with spectroscopic analyses of PFOS and PFOA binding to Mb. Using synchronous fluorescence experiments, Yang et al. demonstrated that, like PFDA, PFOS significantly changed Mb’s heme environment [76]. This study also supports the conclusion that PFOA does not induce significant structural changes near the heme group. Taken together, these findings imply that both the headgroup identity and the length of the hydrophobic tail of PFAS are important factors that can alter Mb structure around its heme group. Additionally, this study demonstrated that PFOS binding increases heme release into solution, implying that PFOS destabilizes Mb’s native structure. Sulfonate’s propensity for stronger electrostatic interactions is a possible explanation as to why PFOA shows an insignificant effect compared to PFOS.

Hb is an α_2_β_2_ heterotetramer that is paralogous to Mb and serves as an oxygen carrying protein. Hb has four heme groups, and therefore four different oxygen binding sites. This structure allows for an allosteric, cooperative oxygen binding mechanism that is critical for oxygen transport and release in many living organisms. Allosteric mechanisms are also susceptible to allosteric effectors that can alter structures, binding affinities, and function.

**Figure 3:**
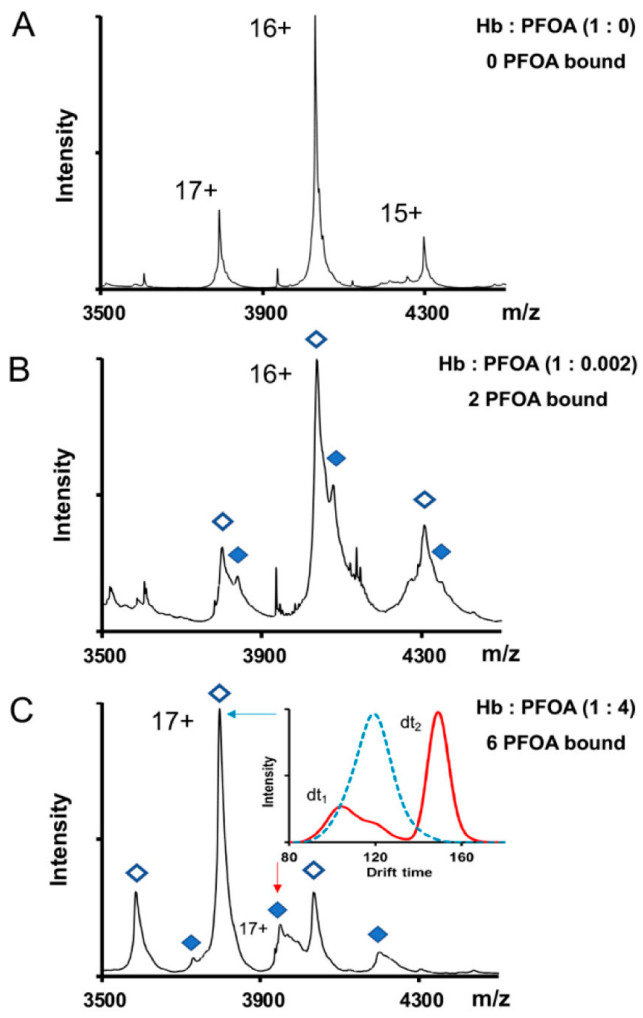
Native mass spectrometry (MS) revealed impact of PFOA on hemoglobin (Hb) conformational ensemble. (**A**) Hb unbound; (**B**) Hb with two bound PFOA molecules. The open diamonds indicate no PFOA bound species, while the closed diamonds indicates PFOA-bound species; (**C**) Hb with six bound PFOA molecules. The +17 Hb charge state is indicated by arrows with the blue arrow showing no PFOA binding and the red arrow showing the species with 6 PFOA molecules bound. Inset: Ion mobilogram showing two different conformations of the alpha subunit with PFOA bound (red trace) compared to PFOA unbound (blue trace). With permission from Trousdale et al. [15].

The heme environment of Hb in the presence of PFAS has been well characterized with fluorescence [74,76], UV-Vis spectroscopy [59,63,64] and MS [15,77]. Hb shows a similar overall increase in intrinsic fluorescence when PFDA and PFOS is present [74,76], implying that these long-chain PFAS disrupt quenching of the aromatic amino acids near heme units by inducing conformational changes. Since Hb has more than one heme environment in its structure, these general findings lack specificity regarding protein structure around individual hemes. These studies also show that PFOS changes Hb’s electronic structure and weakens the ability to retain heme [74,76]. These conclusions are supported with other studies showing that PFOS acts as a structure destabilizer [30]. One important observation from the Qin et al.’s and Di Yang et al.’s studies is that PFOA and PFPA have little impact on Hb fluorescence [74,76]. This finding is inconsistent with more recent Hb studies in which similar optical methods demonstrated structural changes and protein unfolding in the presence of PFOA [78]. Differences in experimental variables such as excitation wavelength, lamp slit widths, or species of Hb are noted and could explain inconsistencies between studies. MS- and time-resolved tryptophan fluorescence have also shown that PFOA generally destabilizes Hb structure [15,77]. Specifically, PFOA has a preference to target the alpha subunit, inducing unique conformational changes that were identified with native and ion mobility MS (Figure 3C). Individually, these results illustrate how different PFAS headgroups and tails affect the heme group in Hb. Collectively, the inconsistent results from study to study suggest that mechanisms describing PFAS–Hb interactions will depend on a host of variables.

Studies investigating PFAS binding stoichiometry have also lead to ambiguous results. Titration experiments monitoring Hb fluoresence suggested at least two binding sites for PFOA [78]. Other reports claimed that both PFOS and PFOA bind to Hb as 1:1 complexes, with a 10-fold excess of each PFAS species in solution [77]. Additionally, complex formation has been observed with lower concentrations of PFOA [15]. This MS data showed that at higher PFOA concentrations, up to six PFOA molecules are bound to Hb by comparing the mass spectra of protein in the presence and absence of PFOA (Figure 3A,B). However, when PFOA concentrations are 500-fold less than the protein concentration, the results showed that only two PFOA molecules are bound to Hb (Figure 3A,C). These latter results showing two PFOA molecules binding are consistent with reports from Perera et al. [78], but may disagree with MS-based experiments that up to six PFOA molecules can be bound to Hb [77]. *In silico* experiments have successfully docked PFOS with Hb [30,76]. These studies claim the most favorable docking position for PFOS is a central cavity of the Hb tetramer. In this case, both hydrophobic and electrostatic interactions are possible, reemphasizing the significance of headgroup and tail length impact on protein structure. The main conclusion from these binding studies is the potential for multiple binding sites on Hb, but the specific site(s) or mechanism remains unknown.

Despite uncertainty regarding PFAS binding mechanisms, binding constant experiments provide some clarity about PFAS affinity for Hb. Dissociation constants were calculated for two PFOA molecules binding to Hb with an uncoordinated heme group. After one PFOA became bound, a second binding PFOA showed much lower affinity, emphasizing the allosteric nature of Hb [78] (Table 1). Additionally, the authors discovered that if the heme was previously coordinated with a CO molecule, only a single PFOA binding site was evident, and this site was much less likely to bind PFOA. This finding suggested that CO near the heme site restricts further binding of participating molecules, indicating competitive inhibition near the heme binding site, with consequences for protein function. In this discussion of binding affinities, Trousdale et al. proposed that PFOA could also induce the loss of its essential cofactor, heme [15]. Using MS, the authors reported an increase in free heme in solution and a broadened charge state distribution, indicating destabilization and a weaker cofactor affinity for the protein. Whether this result is an allosteric effect or simply a consequence of PFOA-induced Hb instability remains unanswered. However, the combination of results outlined above shows that PFAS can have divergent effects on protein structure and binding affinities. Furthermore, inconsistencies between these different studies demonstrate that significant work remains to determine simple questions, including how many PFAS monomers bind to Hb, where these monomers bind, and to what extent PFAS binding affects Hb function.

Cytochromes are broadly defined as membrane-associated hemoproteins, with the principal function of promoting electron transfer. Cytochromes are involved in a diverse range of applications, with varying structures tuned for their function. Cytochromes are categorized into many different super families based either on the characteristics of their heme or on their individual function. The most examined cytochromes in PFAS–protein-related research are cytochrome c (CYT-C) and cytochrome p450 (CYP). The CYT-C family contains a C heme group, which is covalently attached to its polypeptide via two thioester bonds [81]. In humans, CYT-C is an essential water-soluble protein in the electron transport chain. CYP contains a B heme group [82] but is most familiarly categorized in its own superfamily of monooxygenases that catalyze the incorporation of a single diatomic oxygen into a substrate. CYP is further designated based on how electrons are delivered to their catalytic site. In humans, CYPs catalyze reactions important in drug metabolism [83]. Cytochrome substrates often contain sulfonic acid or carboxylic acid moieties that share chemical similarities to the headgroups of PFOS and PFOA, respectively. Competition between native substrates and PFAS molecules has dire implications for human health, specifically liver disease pathogenesis [84,85,86,87]. Thus, current studies examining PFAS effects on cytochromes are largely in pursuit of structural and toxicodynamic data to elucidate inhibition mechanisms.

**Figure 4:**
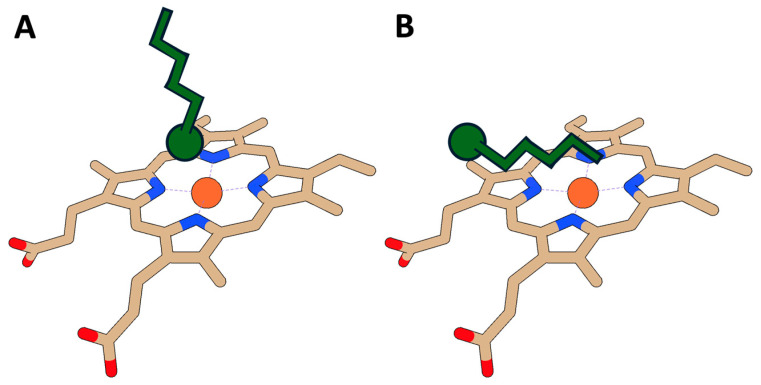
Cartoon representing PFAS binding modes to the heme prosthetic group. CYP3A7 (PDB: 7MK8) active site (orange) with dynamic PFAS (green) binding modes. (**A**) Perpendicular binding conformation of PFAS at low concentrations and (**B**) parallel binding conformation of PFAS at saturated concentrations. Adapted from Hvizdak et al. [79].

One study focused on how PFAS structures affected the CYP heme environment and binding. First, UV-Vis spectroscopy analyzed the heme group region of the CYP3A7 protein with six different PFAS molecules (PFOA, PFOS, GenX, perfluorononanoic acid (PFNA), perfluorononanesulfonic acid (PFNS), and perfluorohexanesulfonic acid (PFHxS)) [79]. CYP3A7 is responsible for important reactions involved in fetal development. Four of the six PFAS molecules induced differences in the absorbing region seen in the heme Soret spectrum [88], indicating a significant conformational change and coordination to the heme group. GenX and PFNS did not lead to changes in heme absorbance. The spectral differences also showed two distinct binding modes as the protein became more saturated with each respective PFAS compound. Molecular docking studies were done to support this mechanism between the heme group and different PFAS (Figure 4). One conformation featured dominating electrostatic interactions with the headgroup near the heme group in a perpendicular fashion (Reverse Type I) (Figure 4A). The other binding conformation showed the tail group lying horizontal to the heme group (Type I) (Figure 4B). This emphasized how the heme group is capable of more than one binding mode. Moreover, there is weak evidence that the sulfonate headgroup in Reverse Type I binding lies closest to the heme iron. Another cytochrome structural study used MS to observe the formation of a protein–PFOA complex in CYT-C. However, this study showed no evidence of PFOA binding to CYT-C [77]. Inconsistencies between studies could suggest weaker affinity of PFOA to CYT-C or simply highlight the difficulty of comparing results with different methodologies.

PFAS structures demonstrate unique affinities and inhibition pathways for CYPs depending on their unique chemical structures (Table 1). The Hvizdak group showed that from their four PFAS candidates, those with longer fluorinated chains and sulfonate headgroups showed the strongest affinity for CYP3A7 in both binding modes [79]. This is consistent with their simulated results, which showed sulfonate groups to be closest to the heme iron (Figure 4A). This result is likely because of stronger electrostatic interactions with sulfonate compared to carboxylate headgroups. The authors noted a decrease in affinity as the PFAS titrant became saturated. They claimed this result demonstrates negative cooperation between PFAS and CYP. In another study, four different CYP (CYP2E1, CYP2D6, CYP3A4, and CYP2C19) enzymes were evaluated for toxicodynamic parameters in the presence of eight different PFAS molecules (PFBS, perfluoroheptanoic acid (PFHpA), PFHxS, perfluorohexanoic acid PFHxA, PFOA, PFOS, PFDA, and PFNA) over a PFAS concentration range of 10 to 100 µM [89]. These CYPs are isoforms involved in xenobiotic metabolism. Calculated K_m_ values showed no obvious pattern that depended on PFAS structure. However, this study claimed that trends of overall inhibition depend on PFAS structure and CYP species. They noted that long hydrophobic tails demonstrated stronger inhibition for tested CYPs. This result is consistent with binding affinity studies on CYP3A7 because K_D_ values decrease as tail lengths increase, implying that a longer tail facilitates a stronger interaction between PFAS and hemoprotein (Table 1). Lastly, the researchers noted competitive, non-competitive, mixed, and atypical inhibition mechanisms. The mechanism differed between different isozymes of CYP, PFAS type, and PFAS concentrations.

The current research clearly shows that PFAS molecules directly impact hemoprotein structure, although specific details of how these impacts occur and their functional implications remain unresolved. Interactions are characterized by changes to the heme environment, number of bound PFAS monomers, PFAS–protein affinities, and allosteric and active site competition. Among all hemoproteins, the heme prosthetic group is unanimously targeted by PFAS. It is vulnerable to a variety of structural changes and destabilization. In general, sulfonate headgroups and longer tail lengths have the largest impact because of their stronger electrostatic and hydrophobic interactions, respectively. However, when all types of PFAS that form complexes with hemoproteins are considered, no apparent trends emerge. Most importantly, there is a lack of information describing how these interactions translate to functionality. To gain an improved understanding for the implications of PFAS, future studies should prioritize the combination of structural findings with functional assays.

## 5. Nuclear Receptors

Nuclear receptors (NRs) are ligand-activated transcription factors that undergo conformational changes upon binding to ligands. Humans have 48 different NRs whose dysfunction has been linked to a wide range of conditions, from prostate cancer [90] to chronic liver diseases [91]. The mechanism of NRs can vary between proteins but can include binding of a ligand, dimerization, binding to coeffectors, and binding to DNA to modulate gene expression. NR structure is highly dynamic and can also undergo substantial conformational change upon ligand binding. Because of this, NR function is highly dependent on the innate stability of the proteins’ secondary structure. Since PFAS have been shown to alter protein stability and structure [15,16], the impact these PFAS have on NRs, especially at low PFAS concentrations, is a critically relevant topic for toxicodynamic research.

Peroxisome proliferator-activated receptors (PPARs) are a superfamily of NRs that have roles in many physiological functions. This superfamily is divided into three subtypes: α, β/δ, and γ. PPARα is primarily involved in the β oxidation and transport of fatty acids [92]. PPARβ/δ is broadly involved in fatty acid metabolism, while PPARγ is involved in regulation of adipocyte differentiation and insulin response. The dynamics of the PPAR protein superfamily have been studied by many groups due to its complex interaction with numerous ligands that coordinate differently to the protein. Depending on the coregulator bound to a specific PPAR receptor, the conformer and dynamics of the protein can dramatically change [93]. The literature has described in detail how PFAS binding results in agonism of PPARs [94].

Binding affinities for PFAS binding to PPARs have been derived using experimental, molecular docking, and molecular dynamics experiments. Examining PPARγ binding to PFOA, K_D_ values have been determined to be 0.057 ± 0.027 µM (Table 1) using equilibrium dialysis [80]. Other short-chain PFAS have shown binding constants in the low micro-molar to sub-micro-molar range. These values show that biologically relevant concentrations of PFAS may bind to PPARs and impact the structure of these proteins. A review of published experimental K_D_ values by Khazaee et al. suggest that the carboxyl headgroups bind more strongly than the sulfonate headgroup (Table 1) [80]. A different *in vitro* binding analysis performed by Zhang et al., however, shows that sulfonic acid PFAS bound tighter than their carboxylic acid counterparts to PPARy [95]. These findings also showed an increase in binding affinity with increasing carbon tail length of up to 11 carbons, after which a slight decrease in affinity was observed. These differences in trends may be due to differences in experimental design, with Zhang et al. using fluorescence displacement and Khazaee et al. using equilibrium dialysis to measure binding affinities. Computational predictions of PFAS binding energies are in good agreement with those calculated by Zhang et al. [96]. Although PFAS with varying chain length have not been examined for PPARγ using equilibrium dialysis, *in vitro* experimentation has confirmed that increasing the length of PFAS generally increases binding affinity [95]. This has been confirmed for many subtypes of PPARs using molecular docking [97] and machine learning [98] simulations as a general trend. For both PPARα and PPARβ/δ, trends in headgroup binding affinity appear similar to PPARγ; however, these trends are difficult to validate with currently published experimental data (Table 1).

**Figure 5:**
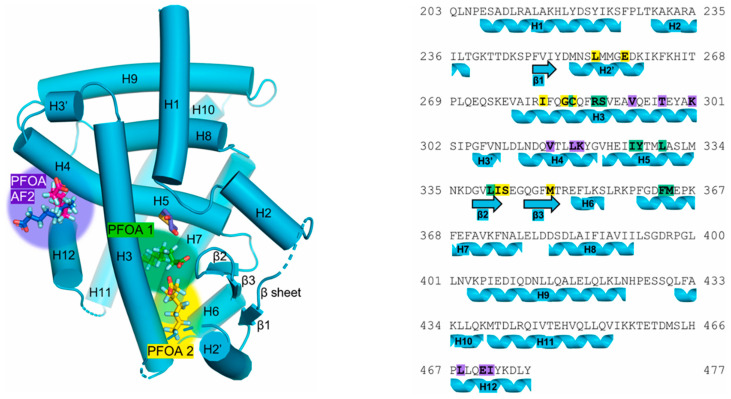
Crystal structure of PFOA in PPARγ ligand binding domain. **Left**: Structure of the PPARγ LBD (cyan) complexed with PFOA. The three sites of PFOA binding are depicted using stick structures. PFOA 1, PFOA 2, and PFOA AF2 are depicted in green, yellow, and purple, respectively. **Right**: PPARγ LBD primary and secondary structure with highlighted PFOA binding sites in the respective color. Image used from Pederick et al. [38].

X-ray crystal structures have shown that a complex forms between the ligand binding domain (LBD) of PPARγ and PFOA [38]. PFOA was shown to occupy three different sites of the LBD (Figure 5). Two of the binding locations were in the ligand binding portion (PFOA 1 and PFOA 2), and the third was present in the coactivator binding site of the LBD (PFOA AF2). Interestingly, PFOA 1 and PFOA 2 do not coordinate the alpha helix in a matter characteristic of full PPARγ agonists such as rosiglitazone. PFOA 1 binds to PPARγ with the fluorinated alkyl chain facing towards this alpha helix, with hydrogen bonding in a manner similar to partial agonists such as nTZDpa and MRL-24. Similarly, bound PFOA 2 stabilizes the β-sheet near the LBD (H2′ and H3), consistent with crystal structures of PPARγ, with partial agonists bound. Molecular dynamics performed on human [96] and Atlantic cod PPARγ [99] showed similar results.

Molecular docking simulations have shown that both PFOA and PFOS can bind to orthosteric and allosteric sites at the same time [100]. Additionally, other groups have shown that PFAS can form a complex with the PPARγ-DNA complex [101]. Upon binding to the complex, the hinge region of PPARγ is predicted to become more dynamic. Another region most affected by PFAS binding was the omega loop, which is thought to be essential for PPARγ allosteric activation. Using molecular dynamics, PFAS that have between 6 and 14 fluorinated carbons in their chains form hydrogen bonds with Tyr 473, His 449, and His 323, residues that have been proposed previously as being important for PPARγ activity [96]. Interestingly, PFOS appeared to have higher affinity for the PPARγ–DNA complex in contrast to the trends reporting higher affinity of PFOA for the PPARγ monomer [80]. These crystallographic and molecular docking and molecular dynamics studies have provided a valuable starting point to consider how PFAS structure leads to agonism of PPARs.

Other NRs have been probed to examine the impact of PFAS on protein function and dynamics. Androgen receptors (ARs) are a category of NRs that bind to androgens (testosterone and dihydrotestosterone). Like PPARs, ARs contain an LBD, a DNA binding domain, and a regulatory domain [102]. ARs are involved in diverse regulatory functions, such as reproductive development, cardiovascular function, and neural systems [103]. Recent *in vitro* investigations have shown that the presence of PFAS can lead to endocrine disruption and alterations in gene expression by these receptors [104]. This result is achieved through an apparent competitive binding mechanism with ARs. Molecular docking simulations from this same group have shown that PFAS’ fluorinated alkyl chain binds key residues in the LBD of ARs, suggesting a potential mechanistic explanation.

Orphan nuclear receptor 4A1 (NR4A1) is an NR that has diverse roles including prevention of fibrosis [105], inflammation, and cellular differentiation [106]. *In vitro* methods have shown that PFAS bind to, change the protein dynamics of, and act as an antagonist for NR4A1 [107]. Upon binding to PFOS, NR4A1 becomes more rigid, which diminishes protein function. This mechanism has been suggested to increase cancer cell proliferation, as the presence of PFOS led to a twofold proliferation of cancer cells compared to control condition.

Together, these current studies have demonstrated that PFAS have the potential to have wide-ranging structural and physiological interactions with the NR class of proteins. Despite the self-evident threat posed by PFAS on protein structure, challenges remain in accurately predicting physical characteristics such as binding affinity of PFAS. Both *in vitro* and *in silico* work have demonstrated that PFAS bind at biologically relevant concentrations. Additionally, it has been described that PFAS can act as agonists or antagonists of NRs. Future work should prioritize optimizing these methods and validating *in silico* structural studies with experimental methods.

## 6. Membrane Receptors

G protein-coupled receptors (GPCRs) constitute the largest family of membrane proteins, and GPCRs regulate most cellular responses to hormones and neurotransmitters. This family of membrane proteins is known to possess seven membrane-spanning α-helical segments separated by alternating intracellular and extracellular loop regions. They are one of the most important means of communication between the internal and external environment of cells and have been the target to many pharmaceutical drugs [108,109]. The major role of GPCRs is to bind agonists and activate specific heterotrimeric G proteins that modulate downstream effector proteins. Several ligands, including biogenic amines, peptides, hormones, glycoproteins, lipids, nucleotides, and ions, bind to GPCRs [110,111] and activate downstream signaling pathways such as the mitogen-activated protein kinase [112].

**Figure 6:**
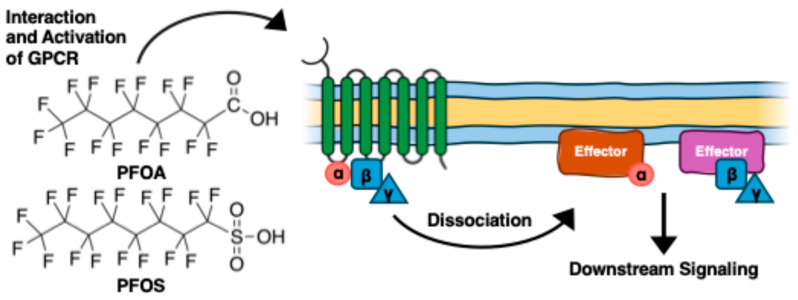
Interaction of PFOA and PFOS with G protein-coupled receptors (GPCRs) and their downstream effects. Both PFOA and PFOS interact with membrane proteins such as GPCR’s, leading to receptor activation. Activation of GPCR leads to the dissociation of the heterotrimeric G protein into α and γβ subunits. This activation triggers binding to effectors and results in downstream signal transduction pathways.

**Figure 7:**
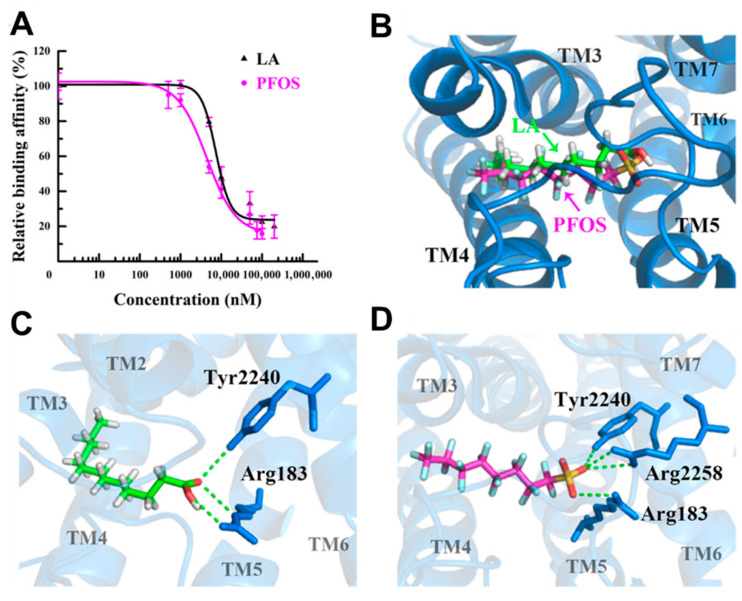
Interaction of lauric acid (LA) and PFOS with human G protein-coupled receptor 40 (GPCR40) by molecular docking and competitive binding assays. (**A**) GPCR40 competitive binding curves with LA and PFOS depicted in black and magenta, respectively; (**B**) molecular docking simulation of PFOS and LA in the binding pocket of GPCR40; (**C**) interaction of LA with select residues of GPCR40; (**D**) interaction of PFOS with GPCR40. GPCR40 is depicted in marine blue, and LA and PFOS are shown as sticks and colored by atom (C: magenta or green, O: red, H: white, F: cyan, S: yellow). Hydrogen bonding is depicted with green dashes. Adapted with permission from Qin et al. [113].

Several studies have investigated how PFAS can affect membrane receptors. PFAS acts as agonists to GPCRs (Figure 6) [113,114,115]. Qin et al. showed that PFOS acts as an agonist for G protein-coupled receptor 40 (GPCR40) in mouse pancreatic β cells, increasing the activity of insulin release in mouse islet β cancer cells (β-TC-6) cells by raising intracellular calcium levels [113]. These variations in insulin production and release of calcium in β-TC-6 cells suggest how PFOS can alter the physiological processes upon binding to GPCR40 (Table 2). Fluorescence competitive binding and docking simulations revealed PFOS binding in human GPR40. Through hydrogen bond formation with key amino acid residues, the simulations showed both PFOA and PFOS oriented toward the inner part of GPCR40 (Figure 7). Given that PFOS has been shown to interact with human GCPR40, this mechanism may be the first step toward disruption of insulin production in humans.

Guan and colleagues reported the binding of PFOA to G protein-coupled estrogen receptor (GPER) [114]. In their study, molecular dynamics simulations and fluorescence competitive binding analyses revealed that PFOA caused similar conformational changes and signaling responses to GPER as its natural agonist, G1. In both cases, ligand binding caused a stabilization of the protein structure; however, near the protein’s active site, PFOA-induced changes differed from patterns induced by G1. Moreover, downstream signaling pathways of GPER, as modulated by PFOA, were determined through transcriptomic and proteomic analysis, thus revealing the causal relationship of the disrupting effects of PFOA binding to GPER. Liang et al. also studied the binding of PFAS (PFHS, PFOS and PFBS) with GPER using molecular docking, molecular dynamics simulations, and spectroscopic analysis [116]. The studies showed that PFAS binding to GPER stabilized the protein structure and altered secondary structural components, which was consistent with their Fourier transform infrared spectroscopy and three-dimensional fluorescence results, indicating possible receptor regulation. This suggests that the activation of GPER by PFAS could have similar effects to those induced by known GPER agonists and downstream signaling pathways [114].

Both PFOA and PFOS have also been shown to act as non-competitive antagonists to gamma-aminobutyric acid (GABA_A_) receptor [115]. The GABA_A_ receptor serves as the primary inhibitory neurotransmitter receptor in the central nervous system and is vital for brain development. An inhibitory electrical signal known as GABA-evoked current is created when GABA binds to GABA_A_ receptors, opening chloride channels that lower neuronal excitability and lessen the possibility of an action potential. Both PFOS (LOEC 0.1 µM, IC50 0.28 µM) and PFOA (LOEC 10 µM, IC50 22 µM) reduced the GABA_A_-evoked current in a concentration-dependent manner. In the study, inhibition of the GABA_A_-evoked ion current caused by PFOS was largely irreversible compared to that of PFOA, which was rapidly reversible. Moreover, PFOS at 100 µM led to higher spike and burst rates, indicating mild hyperexcitation, while PFOA showed minimal impacts in rat cortical cell cultures [115]. These findings highlight how PFAS such as PFOA and PFOS can exert disruption of broad physiological processes upon binding to both GCPR and GABA_A_ proteins (Figure 6 and Figure 7), and how these compounds can alter endocrine and neural regulation.

## 7. Knowledge Gaps

This manuscript reviewed the current literature of protein–PFAS interactions, focusing on albumins, hemoproteins, and nuclear and membrane receptors. These studies consistently report that PFAS binding to proteins causes structural changes that may lead to altered function. While this establishes a foundational knowledge base of PFAS–protein interactions, there still is much to be learned.

Binding affinities are important, because they serve as an indicator as to how strongly a PFAS binds to a protein, and can be compared to endogenous ligand affinities. There is a lack of experimentally derived binding affinities for a wider set of proteins. Although some values are available (Table 1 and Table 2), reported results sometimes have high variability. Additionally, the lack of consistent approaches for determining binding affinities makes inter-protein comparisons subject to uncertainty. A standardized approach to obtaining quantitative binding affinities across a variety of protein systems and PFAS species (systematically varying headgroup and tail length) would provide more insight and some predictive power for assessing PFAS effects on protein structure and function.

With over 15,000 PFAS compounds [1], many groups have used *in silico* approaches to determine how strongly a wide range of PFAS binds to a set of proteins. However, this approach also can lead to difficulties comparing trends across different protein classes. PFAS exhibit wide structural diversity in chain length and headgroup chemistry, making accurate force-field parameterization challenging. PFAS’ amphiphilic nature also requires simulations to explicitly include water and lipid molecules to capture partition and binding behavior, greatly increasing system size and computational complexity. Computationally modeling PFAS interactions with membrane-embedded proteins such as GPCRs can be difficult, as PFAS interact with membranes in addition to binding to the proteins themselves. Computational studies can also be challenging when calculating changes in protein dynamics for allosteric systems like Hb and NRs. Finally, as previously described, comparing results across different *in silico* studies poses challenges due to studies using differing parameters, such as the presence of water molecules (and how much water) and limiting PFAS binding to certain regions of the proteins where calculations converge to metastable structures. However, computationally studying PFAS interacting with proteins remains a useful method to screen dozens to hundreds of PFAS–protein systems efficiently. Testing these models with quantitative experimental data should remain a priority in the coming years.

To supplement binding affinity studies, many computational studies have examined the location of PFAS bound to proteins. Simulation techniques such as molecular docking and molecular dynamics provide valuable information on potential PFAS binding sites in proteins. Additionally, as we have described, simulation results have suggested that headgroup composition can change the orientation of PFAS in proteins such as Hb [79], leading to differential structural destabilization. To gain further insight on this, structural biology approaches such as X-ray crystallography or cryogenic electron microscopy have begun providing the detailed structural information needed to validate the *in silico* predictions. Obtaining high-resolution structures of PFAS molecules bound to more protein structures will continue to reveal the subtle variables that dictate the number of PFAS that bind to proteins and provide insight into how these PFAS disrupt or alter function.

Molecular dynamics simulations have also predicted that PFAS can alter the dynamics and structure of globular proteins generally [16]. This finding is especially relevant to highly dynamic or allosteric proteins like NRs and Hb and could provide a more nuanced insight into mechanisms that render PFAS so dangerous to humans. Future experimental work using techniques that probe the protein dynamics (hydrogen deuterium exchange, ultrafast emission and transient absorption, etc.) or computational work using solved protein structures would provide valuable data on the changes in protein dynamics or conformation, which could explain the mechanism of PFAS toxicity.

Examining the impact of PFAS–protein interactions is an ongoing and increasingly important topic. A critical challenge, however, is the ability to screen the immensely large library of PFAS against the catalogue of proteins *in vivo*. Both *in silico* and *in vitro* experimentation have begun to tackle this challenge, but the large variability in approaches and the wide array of PFAS–protein combinations is daunting. A considerable effort should therefore be spent confirming that results from *in vitro* and *in silico* approaches hold true to proteins *in vivo*. One avenue by which this can be achieved is by gaining a better understanding of the mechanisms by which different proteins bind to, or have their dynamics disrupted by, PFAS. These results should then be compared to the PFAS-implicated human health conditions that are already well established in the literature.

## 8. Conclusions

Systematic reviews and meta-analyses estimate that diseases attributable to PFOA and PFOS cost the USA between USD 5.5 and USD 62.6 billion [117]. PFAS compounds are globally distributed and environmentally persistent. Understanding the molecular underpinnings of PFAS pathology is a global and enduring concern. One avenue available for PFAS to adversely impact human health is through protein: PFAS interactions. To date, research into these interactions has been focused on albumins, hemoproteins, nuclear receptor proteins, and membrane receptor proteins. These classes of proteins are involved in essential biological processes, including transport, respiration, electron transport, metabolic processes, and regulation. From the current literature, we can glean some insights into protein:PFAS interactions.

PFAS molecules bind to protein and protein complexes with different affinities depending on the protein and PFAS. When taking the same chain length into account, the sulfonated PFOS had lower IC50 for GABA receptor and higher inhibition for cytochromes than the carboxylated PFOA [115], yet the inverse was true for PPARγ [80]. Interestingly, molecular dynamics simulations predict that when PPARγ is complexed with DNA, the sulfonated form has greater binding affinity [101]. In hSA, the Sudlow’s II drug binding site had increased specificity towards carboxylated PFAS [28]. The strength of interactions can also be similarly influenced by the length of the fluorinated alkyl tail, which also is not held as a universal trend. In hSA, Sudlow’s I drug binding site favored shorter-chain PFAS, while higher affinity towards longer-chain PFAS was observed in the tryptophan site [28]. Additionally, longer tail lengths in PFAS increased inhibition in cytochromes [89] and affinity for PPARs generally [95,97,98]. The PFAS length and headgroup has a degree of influence on its ability to bind to different proteins, making it exceptionally difficult to generalize trends across protein systems.

PFAS amphiphilicity endows these compounds with the ability to bind to proteins through several mechanisms, all of which can lead to protein structural alterations and changes in conformational ensembles. PFAS compounds have been shown to cause structural changes in albumins like BSA and hSA, Hb, ARs, NR4A1, and GPER. These alterations vary depending on the protein, PFAS, and potentially the presence of additional ligands. For BSA, five PFAS compounds bind in the hydrophobic pockets of the protein, creating an even more hydrophobic environment [65]. In the other albumin, hSA, binding of PFOS molecules caused the structure to take on a more compact conformation in the absence of myristic acid [36]. When myristic acid was also present, PFOS caused an elongation of hSA, while no structural changes were observed when PFOA was bound [37]. PFOA caused unfolding/destabilization in Hb [15,78]. For the nuclear receptors PPARγ and NR4A1, PFAS generally caused a stiffening of the protein structures [38,96,107], except when PPARγ was complexed with DNA, a loosening of a hinge region was predicted with molecular dynamics simulations [101]. Additionally, in the membrane receptor GPER, PFOA caused displacement of transmembrane regions [114]. Interestingly, PFAS bind to proteins in a variety of places, as exemplified in the crystal structures of PPARγ with PFOA, where PFOA was present in various places with different orientations (Figure 6).

Together, these findings reveal that PFAS compounds bind to proteins through a number of mechanisms, which can lead to structural alterations and disruption of allosteric networks. This makes PFAS uniquely deleterious to various classes of proteins. The non-uniformity in binding preference might be due to the lipophilic/amphiphilic character and relatively high flexibility of the fluorinated carbon chain, which may lead to specific individual PFAS–protein pairings. Therefore, the use of many experimental and computational approaches is required to comprehensively study the complex toxicodynamic mechanisms that these molecules use to impact overall human health.

## Figures and Tables

**Table 1:** Summarized experimentally derived binding constants determined for Cytochrome p450 3A7 (CYP3A7), hemoglobin (Hb), and three peroxisome proliferator-activated receptors (PPARs). The type of protein, PFAS, K_D_ values, and the study are denoted in the columns from left to right. Proteins that did not have a K_D_ value reported are denoted with “N/A”.

Protein	PFAS	K_D_ (µM)	Study
CYP3A7	PFOA Reverse Type 1	237.5 (209.7–286.2)	[79]
	PFOA Type 1	477.9 (369.9–727.7)	
	PFOS Reverse Type 1	61.74 (50.72–86.71)	
	PFOS Type 1	90.39 (66.38–170.0)	
	PFNA Reverse Type 1	76.87 (67.68–87.41)	
	PFNA Type 1	181.2 (151.4–241.4)	
	PFHxS Reverse Type 1	296.8 (277.8–319.1)	
	PFHxS Type 1	731.3 (539.9–1283)	
Met-Hb	PFOA (single binding)	0.8	[78]
Met-Hb	PFOA (double binding)	630	
CO-bound Hb	PFOA	139	
PPAR-α	PFBA	N/A	[80]
	PFHxA	0.097 ± 0.070	
	PFHpA	N/A	
	PFNA	0.083 ± 0.028	
PPAR-δ	PFBA	0.044 ± 0.013	
	PFBS	N/A	
	PFHxS	0.035 ± 0.0020	
	PFOS	0.69 ± 0.33	
PPAR-γ	PFOA	0.057 ± 0.027	
	PFOS	8.5 ± 0.46	
PPAR-γ	PFBA	N/A	[39]
	PFHxA	N/A	
	PFHpA	1330.4 ± 119.0	
	PFOA	300.9 ± 36.2	
	PFNA	155.4 ± 7.7	
	PFDA	84.4 ± 7.6	
	PFUnA	58.2 ± 11.8	
	PFDoA	143.1 ± 14.8	
	PFTeDA	157.8 ± 15.0	
	PFHxDA	128.2 ± 19.9	
	PFOcDA	107.6 ± 34.6	
	PFBS	N/A	
	PFHxS	285.3 ± 47.1	
	PFOS	93.7 ± 8.3	
	6:2 FTOH	N/A	
	8:2 FTOH	N/A	

**Table 2:** Experimentally derived IEC50 and relative binding affinity (RBA) values determined for GPCRs. The type of protein, PFAS, IEC50, RBA, and the study are described in columns from left to right. Proteins that did not have an IECE50 or RBA value are denoted with “N/A,” and not-reported values are denoted with “N/R”.

Protein	PFAS	IEC50 (µM)	RBA	Study
GCPR40	LA	7.4 ± 0.6	1	[113]
	TAK-875	<0.1	616.7	
	PFBA	N/A	N/A	
	PFBS	N/A	N/A	
	PFHxA	N/A	N/A	
	PFHxS	167.7 ± 9.8	<0.1	
	PFHpA	N/A	N/A	
	PFOA	119.3 ± 19.3	0.1	
	PFOS	4.4 ± 0.7	1.7	
	PFNA	24.3 ± 18.5	0.3	
	PFDA	5.0 ± 1.0	1.5	
	PFUnA	2.9 ± 0.6	2.6	
	PFDoA	0.7 ± 0.1	10.6	
	PFTriDA	N/A	N/A	
	PFTeDA	N/A	N/A	
	PFHxDA	N/A	N/A	
	PFOcDA	N/A	N/A	
GPER	PFOA	8.34	N/R	[114]

## Data Availability

No new data were created or analyzed in this study. Data sharing is not applicable to this article.

## Abbreviations

β-TC-6	Islet β cancer cells
AR	Androgen receptor
BSA	Bovine serum albumin
CYP	Cytochrome p450
CYT-C	Cytochrome c
DSF	Differential scanning fluorimetry
FTOH	Fluorotelomer alcohol
FQ	Fluorescence quenching
GABA_A_	Gamma-aminobutyric acid receptor
GenX	Hexafluoropropylene oxide dimer acid
GPCR	G-protein-coupled receptor
GPCR40	G-protein-coupled receptor 40
GPER	G-protein-coupled estrogen receptor
Hb	Hemoglobin
hSA	Human serum albumin
ITC	Isothermal titration calorimetry
LA	Lauric acid
LBD LOEC	Ligand binding domain Lowest Observed Effect concentration
Mb	Myoglobin
MS	Mass spectrometry
Myr	Myristic acid
NR	Nuclear receptor
NR4A1	Orphan nuclear receptor 4A1
PFAS	Per- and polyfluoroalkyl substances
PFBA	Perfluorobutanoic acid
PFBS	Perfluorobutanesulfonic acid
PFDA	Perfluorodecanoic acid
PFDoA	Perfluorododecanoic acid
PFHpA	Perfluoroheptanoic acid
PFHS	Potassium perfluorohexyl sulfonate
PFHxA	Perfluorohexanoic acid
PFHxDA	Perfluorohexadecanoic acid
PFHxS	Perfluorohexanesulfonic acid
PFNA	Perfluorononanoic acid
PFNS	Perfluorononanesulfonic acid
PFOA	Perfluorooctanoic acid
PFOcDA	Perfluorooctadecanoic acid
PFOS	Perfluorooctanesulfonic acid
PFPA	Perfluoropentanoic acid
PFTeDA	Perfluorotetradecanoic acid
PFTriDA	Perfluorotridecanoic acid
PFUnA	Perfluoroundecanoic acid
PPAR	Peroxisome proliferator-activated receptor
RBA	Relative binding affinity
